# YAP-Dependent Induction of CD47-Enriched Extracellular Vesicles Inhibits Dendritic Cell Activation and Ameliorates Hepatic Ischemia-Reperfusion Injury

**DOI:** 10.1155/2021/6617345

**Published:** 2021-06-22

**Authors:** Zenan Yuan, Linsen Ye, Xiao Feng, Tian Zhou, Yi Zhou, Shuguang Zhu, Changchang Jia, Haibo Li, Dongbo Qiu, Kun Li, Wei Liu, Yang Li, Hui Tang, Guoying Wang, Qi Zhang, Yang Yang, Guihua Chen, Hua Li

**Affiliations:** ^1^Department of Hepatic Surgery and Liver Transplantation Center, The Third Affiliated Hospital of Sun Yat-sen University, Guangzhou, China; ^2^Guangdong Provincial Key Laboratory of Liver Disease Research, Guangzhou, China; ^3^State Key Laboratory of Ophthalmology, Zhongshan Ophthalmic Center, Sun Yat-sen University, Guangzhou, China; ^4^Department of General Surgery, Guangdong No.2 Provincial People's Hospital, Guangdong Province, China; ^5^Cell-Gene Therapy Translational Medicine Research Center, The Third Affiliated Hospital of Sun Yat-sen University, Guangzhou, China

## Abstract

Hepatic ischemia-reperfusion injury (IRI) is the most common cause of liver damage leading to surgical failures in hepatectomy and liver transplantation. Extensive inflammatory reactions and oxidative responses are reported to be the major processes exacerbating IRI. The involvement of Yes-associated protein (YAP) in either process has been suggested, but the role and mechanism of YAP in IRI remain unclear. In this study, we constructed hepatocyte-specific YAP knockout (YAP-HKO) mice and induced a hepatic IRI model. Surprisingly, the amount of serum EVs decreased in YAP-HKO compared to WT mice during hepatic IRI. Then, we found that the activation of YAP increased EV secretion through F-actin by increasing membrane formation, while inhibiting the fusion of multivesicular body (MVB) and lysosomes in hepatocytes. Further, to explore the essential elements of YAP-induced EVs, we applied mass spectrometry and noticed CD47 was among the top targets highly expressed on hepatocyte-derived EVs. Thus, we enriched CD47^+^ EVs by microbeads and applied the isolated CD47^+^ EVs on IRI mice. We found ameliorated IRI symptoms after CD47^+^ EV treatment in these mice, and CD47^+^ EVs bound to CD172*α* on the surface of dendritic cells (DCs), which inhibited DC activation and the cascade of inflammatory responses. Our data showed that CD47-enriched EVs were released in a YAP-dependent manner by hepatocytes, which could inhibit DC activation and contribute to the amelioration of hepatic IRI. CD47^+^ EVs could be a potential strategy for treating hepatic IRI.

## 1. Introduction

Hepatic IRI occurs when blood flow is restored after a period of hepatic ischemia [1, 2]. Since the donated grafts are often highly susceptible to IRI, while the available organs are in severe shortage, IRI has become one of the main obstacles in liver transplantation [3]. There is an urgent need for developing protective strategies against IRI to promote the survival of patients after liver transplantation.

The mechanisms governing IRI are highly complex and have been the focus of investigation for decades with numerous factors been identified with specific function. Among the principle factors in hepatic IRI, Yes-associated protein (YAP), the key effector of the Hippo pathway, has been reported to be a hinge joint in inflammation and oxidative stress, but its role and mechanism remain unclear. Some reports suggested that YAP played a protective role in the hepatic IRI model [4], while others found that YAP-expressing hepatocytes activated inflammation and aggregated liver fibrosis through the YAP/TAZ/CYR61 axis [5]. These conflicting findings suggested that the mechanisms in YAP's function have not been fully unveiled, since YAP has been found to regulate the binding of actin and angiomotin (AMOT) family members [6], which play an irreplaceable role in endosomal transport and the secretion of extracellular vesicles (EVs) [7]. Here, we investigated the paracrine effect of YAP through the secretion of EVs.

EVs are phospholipid bilayer vesicles widely distributed in body fluids as a form of intercellular communication and modulation of cellular activities in recipient cells. Multiple immunomodulatory effects of EVs have been reported [8, 9], but the mechanism linking hepatic injury to associated immune responses through EVs has not been found. Initiated by hypoxic stress, IRI displayed extensive inflammatory responses that are driven by innate immunity and supported by adaptive immunity [4, 10]. Hepatic DCs act as one of the major mediators in local immune responses [11]. Activated DCs could [12] trigger both innate and adaptive immunity and aggregate local injury. However, reports also showed that hepatic DCs could limit certain inflammation and promote immune tolerance [13]. The immune-modulatory activity of DCs is regulated by the expression level of CD47, the well-known “don't eat me” signal [14]. By binding to the counter-receptor signal-regulated protein alpha (SIRP alpha/CD172*α*), which is mainly expressed on the surface of myeloid cells, CD47 could initiate the inhibitory signaling to restrain inflammation [15, 16].

In this study, we found CD47 on hepatic EVs targeted CD172^+^ DCs and potently inhibited their activation, therefore alleviated hepatic IRI, whereas YAP expression is required for EV secretion of hepatocytes. Mechanistically, YAP induced EV secretion through F-actin by increasing membrane formation, with the inhibition of MVB and lysosome fusion. Our results revealed a novel mechanism for maintaining immune balance in hepatic IRI, via the regulation of YAP-EV-CD47 axis in hepatocyte-DC crosstalk, which suggested a novel therapeutic strategy utilizing CD47^+^ EVs in treating hepatic IRI.

## 2. Methods

### 2.1. Human Subjects

The selected samples were from 69 patients who underwent liver transplantation from donors after cardiac death (DCD) since April 2010 to April 2015 at the liver transplantation center of the third affiliated hospital, Sun Yat-sen University. Cases of 64 males and 5 females were included, with an average age of 47.43 years (21-72 years), and were all treated by modified piggyback orthotopic liver transplantation surgery. The details of the patient demographics are listed in Supplementary Table 1. The selection criteria were as published [17].

### 2.2. Animals

Six to eight weeks of C57BL/6J, BALB/c male mice were obtained from Guangzhou University of Chinese Medicine, China. YAP-HKO mice were constructed by crossing Albumin-Cre (*Alb-Cre*) mice and *Yap^flox/flox^* mice from the Model Animal Research Center of Nanjing University (Nanjing, China). CD11c-DTR mice were purchased from Jackson Laboratory (Farmington, CT, USA). All the animal experiments conducted in this study were approved by the animal ethics committee of the third affiliated hospital of Sun Yat-sen University.

### 2.3. Mouse Hepatic IRI Model

The 70% liver ischemia-reperfusion (I/R) injury model was constructed after 0.6% pentobarbital sodium (100 *μ*L/10 g) was injected intraperitoneally [17]. In brief, the artery/portal vessel was clamped to the cephalad lobes for 90 minutes during ischemia, and reperfusion was performed by loosening the atraumatic vascular clamp. The sham operation group underwent the same operation except that blood vessels were not clamped. Animals received injections of EVs (100 *μ*g/kg b.w. in PBS) or PBS immediately before reperfusion.

### 2.4. Cells and Reagents

Human hepatic L02 cells from the Cell Bank of the Chinese Academy of Sciences in Shanghai were used in the *in vitro* experiments [18]. Antibodies against YAP1 (ab56701, Abcam), Alix (ab186429, Abcam), CD81 (D5O2Q, CST), TSG101 (ab125011, Abcam), *β*-actin (8H10D10, CST), calnexin (ab22595, Abcam), and CD47 (ab175388, Abcam) were used for western blot. When CD47 was blocked, two doses of 100 *μ*g (BE0270, BioXcell) were given intraperitoneally two days before IRI and the same day at IRI. The control group used the same dose of rat immunoglobulin G2a (IgG2a) isotype (BioXcell) [19].

### 2.5. EV Isolation and Analysis

EVs in mouse serum were isolated with an EV isolation kit (SmartSEC Mini EV Isolation System) and detected with an ELISA kit detecting CD81 exosome (EXOEL-CD81A-1) from System Biosciences.

L02 and primary hepatocytes (PMH) were cultured 48 hours at 37°C in serum-free DMEM. The supernatant was collected and centrifuged at 2,000 *g* for 10 min at 4°C to remove the cell debris [20]. Then, after filtering through 0.22 *μ*m filters, the filtrate was ultracentrifuged at 100,000 *g* for 120 minutes in a Beckman SW28Ti rotor. After the resuspension by PBS, the pellets were ultracentrifuged again at 100,000 *g* for 120 min. The final EV pellets were dissolved in PBS for further experiments [20].

To isolate CD47^+^ EVs, a total of 200 *μ*g PMH-EVs were mixed with nonblocking anti-CD47 antibody (REA170)-FITC for 30 min at 4°C. Then, incubated with anti-FITC magnetic beads (Miltenyi Biotec; 1 *μ*L/*μ*g EVs) overnight at 4°C. CD47^−^ EVs and CD47^+^ EVs were separated by magnetic beads, and both supernatants were washed with PBS and pelleted by ultracentrifugation.

EVs were further analyzed by a Micro BCA Protein Assay Kit to determine protein concentrations (Thermo, #23235). Particle diameters and amounts were observed by the NanoSight system (NS300, Malvern, Ranch Cucamonga, CA, USA). Further details of methods were described in supplementary materials (available here).

### 2.6. Real Time-PCR Analysis

Real-time PCR was performed using the following primer sequences [21]: YAP-forward: CCCAGACTACCTTGAAGCCA and YAP-reverse: CTTCCTGCAGACTTGGCATC; CYR61-forward: CTGCAGCAAAACTCAGCCCT and CACAGGGTCTGCCTTCTFAC and CYR61-reverse: CTTCCTGCAGACTTGGCATC; TRX1-forward: ATGGTGAAGCTGATCGAGAGC and TRX1-reverse: GGCATATTCAGTAATAGAGGC; HO-1-forward: GCAGAGAATGCTGAGTTCATG and HO-1-reverse: CACATCTATGTGGCCCTGGAGGAGG; GAPDH-forward: GCGGGAAATCGTGCGTGAC and GAPDH-reverse: CGTCATACTCCTGCTTGCTG.

### 2.7. Histopathology and Immunostaining

Tissues were fixed by 4% paraformaldehyde and embedded in paraffin, followed by slicing with a microtome and staining with hematoxylin-eosin. For the graft biopsy, the immunohistochemical staining results were assigned the mean score considering the product of the intensity of the stain and the percentage of positive cells. 0 is negative, 1 to 4 are mildly positive, 5 to 8 are moderately positive, and 9 to 12 are strong positive [22, 23]. Negatives are included in the low group, while weakly positive, moderately positive, and strong positives are included in the high group. Each section was independently assessed by two pathologists. Frozen liver tissue sections or cells were fixed, blocked according to standard procedures [19]. For immunofluorescence analysis, we used antibody YAP (2F12, Novus), RAB7 (ab137029, Abcam), EEA1 (610456, BD Biosciences) [24], LysoTracker™ Red DND-99 (ThermoFisher), Rhodamine Phalloidin (PHDR1, Cytoskeleton), Cy2-conjugated goat anti-rabbit IgG (111-225-144, Jackson ImmunoResearch), and Cy3-conjugated anti-mouse IgG (AP124C, EMD Millipore).

### 2.8. Plasmid Construction and Transfection

By short hairpin RNA inference, the silenced sh-YAP expression plasmids were purchased from Sigma, with the control group using the nontargeting shRNA expression plasmid (MISSION Plko.1-puro Empty Vector Control Plasmid DNA). The cells were transfected according to standard procedures [25].

### 2.9. Primary Hepatocyte Isolation

The liver was perfused with an immunoenzyme (10 units) to digest connective tissue [19]. Then, use Percoll gradient to purify primary hepatocytes.

### 2.10. Hepatic Lymphocyte Isolation

After the liver tissue was gently crushed, the lymphocytes were obtained using percoll gradient purification [19]. Then, the cell suspension was stained for viability analysis, and markers including CD45, CD47, CD172a, CD11c, I-A/I-E, TNF-*α*, and IL-12 p40 (eBioscience, San Diego, CA).

### 2.11. T Cell Isolation

CD4^+^ and CD8^+^ T cells were isolated from the spleen of experiment mice by CD4^+^ and CD8^+^ T cell isolation kit II (Miltenyi Biotec, Bergisch Gladbach, Germany) and followed by labeling with CFSE (Invitrogen) according to the manufacturer's instructions [26].

### 2.12. DC Function Assay

Bone marrow monocytes were obtained from the cell suspension of mouse tibia and femur [27]. To generate bone marrow-derived DC (BMDCs), 10 ng/mL recombinant mouse granulocyte-macrophage colony-stimulating factor (rmGM-CSF) and 1 ng/mL IL-4 (R&D) were added in the medium (Figure S2C). BMDCs were collected after verifying purity (more than 95% CD11c positive) and 1 × 10^6^/mL cells were incubated under 1 mg/mL LPS stimulation, and the experiment group was supplemented with 30 mg/mL EVs for 24 h, with PBS supplemented as control. To evaluate the antigen-presenting ability of DCs, LPS-stimulated BMDCs were collected and cocultured with CFSE-labeled CD4^+^ and CD8^+^ T cells from BALB/c mouse at a ratio of 1 : 10 for 96 hours.

### 2.13. Cytokine ELISA

Both murine serum and culture supernatants from BMDCs were harvested for further cytokine analysis [28]. ELISA kits to measure IL-12 p40, TNF-*α*, and IL-6 levels individually were purchased and used under the manufacturers' instructions (eBioscience, San Diego, CA).

### 2.14. Immunogold Electron Microscopy

For immune electron microscopy, we used anti-CD47 antibody (Novus-NBP2-44408) for primary staining and incubated overnight at 4°C. Then, we applied 18 nm colloidal gold-conjugated goat anti-mouse IgG (115-215-166, Jackson ImmunoResearch) as the secondary antibodies. The following steps were performed according to previous reports [24].

### 2.15. Statistical Analyses

Student's *t*-test was used to analyze statistical comparisons between groups. The resulting *p* value < 0.05 is considered significant statistically.

## 3. Results

### 3.1. YAP Silencing during I/R Aggravates Liver Damage While Decreases Serum EV (sEV) Concentration

**Figure 1:**
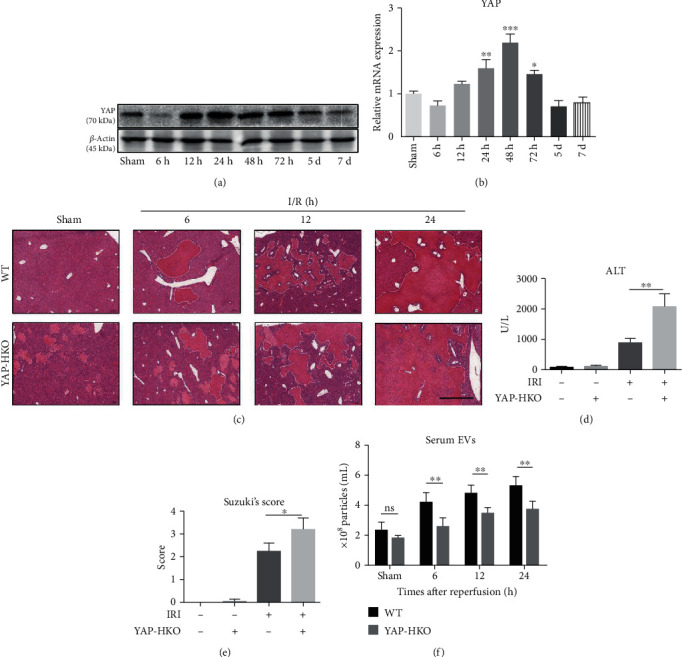
Silencing of YAP exacerbates liver injury and decreases sEVs' concentration during IRI. (a) Western blot analysis of hepatic YAP protein levels of IRI mice at different time points; (b) the mRNA levels of hepatic YAP from IRI mouse liver were detected by qPCR at different time points. (c) H&E staining of ischemic livers in WT and YAP-HKO mice showed that the injury increased with reperfusion time and reached the peak at 24 h after reperfusion. Scale bar, 1 mm. (d) Serum ALT levels of YAP-HKO mice were significantly higher than WT mice with I/R at 24 h (2059 ± 214.2 U/L vs. 872.8 ± 76.15 U/L, ^∗∗^*p* < 0.01). (e) Suzuki's histological score of YAP-HKO mice was higher than WT mice with I/R at 24 h (score: 2.25 ± 0.17 vs. score: 3.2 ± 0.25, ^∗^*p* < 0.05). (f) The concentrations of sEVs from WT and YAP-HKO groups at different time points after IRI, as measured by CD81 exosome ELISA kit. *n* = 6 per group. ^∗∗^*p* < 0.01, ns: *p* > 0.05, *t*-test.

We first confirmed YAP expression level to be negatively correlated with hepatocellular damage in I/R-stressed human orthotopic liver transplantation (OLT) samples (Supplementary Fig. 1A-C, Supplementary Table 1) [4]. And during hepatic IRI in mice, hepatic YAP protein and YAP mRNA gradually increased over time (Figures 1(a) and 1(b)). Then, hepatocyte-specific YAP knockout (YAP-HKO) mice were constructed to study whether YAP knockout could aggregate hepatic dysfunction in hepatic IRI mice (Supplementary Fig. 1D-E), since the pan-tissue YAP knockout mice are lethal in a fetus. By these YAP-HKO mice with I/R at 24 h, we observed severe hepatic damage by pathological analysis (Figure 1(c)) and measured their serum ALT levels (Figure 1(d)), while there were no obvious differences between YAP-HKO and wild-type (WT) mice under sham operation. Likewise, Suzuki's score indicated significantly increased hepatocellular damage by YAP silencing (Figure 1(e)). These data suggested that YAP silencing can exacerbate hepatic IRI.

Next, we isolated sEVs from WT mice and YAP-HKO mice after hepatic IRI with a serum EV isolation kit [29]. By CD81 exosome ELISA kit for quantification, we found that sEVs' concentration gradually increased after IRI and reached the peak in 24 hours (Figure 1(f)). This trend was in consistency with the YAP protein expression levels as shown in Figures 1(a) and 1(b). Silencing YAP in vivo decreased sEVs' concentration after IRI (Figure 1(f)). sEVs were verified by transmission electron microscopy (TEM) (Supplementary Fig. 2A). EV markers including CD81, ALIX, and TSG101 were expressed in sEV lysates, but the endoplasmic reticulum marker calnexin was not found in sEVs [30] (Supplementary Fig. 2B). Our *in vivo* observations indicate that sEVs' concentration is affected by YAP expression, and it is necessary to explore the mechanism and effect of YAP regulation on EV secretion.

### 3.2. YAP Silencing Decreases the Formation of EVs in an F-Actin-Dependent Manner

**Figure 2:**
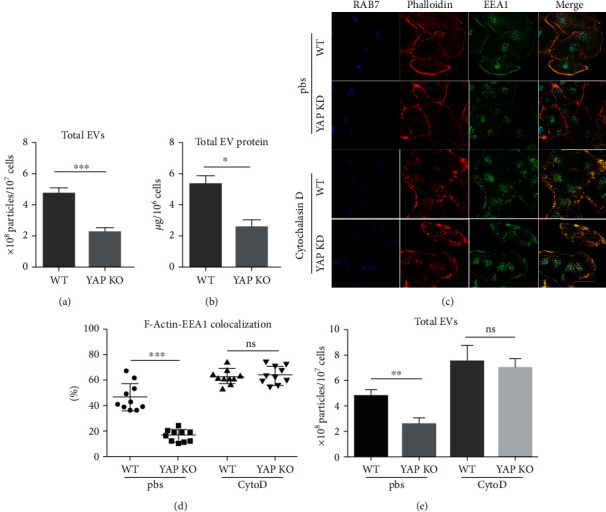
Hepatic YAP silence decreases EV formation. (a) EVs extracted from culture medium of WT or YAP-KD L02 cells were subjected to nanoparticle tracking analysis (NanoSight) for quantitative [20]. *n* = 3, ^∗∗∗^*p* < 0.001, *t*-test. (b) A BCA Protein Assay Kit was used to detect the protein concentration of EVs. *n* = 3. ^∗^*p* < 0.05, *t*-test. (c) Rhodamine-labeled phalloidin staining (labeling membrane-associated F-actin, red), EEA1 staining (labeling early endosomes, green), and RAB7 staining (labeling late endosomes) for YAP-KD and WT L02 cells under either PBS or cytochalasin stimulation. Scale bar, 10 *μ*m. (d) Statistical analysis of amounts of EEA1 that colocalized (orange) with F-actin, shown as percentages of the total EEA1, *n* = 10, ^∗∗∗^*p* < 0.001, ns: *p* > 0.05, *t*-test. (e) EVs extracted from a culture medium of equal amount of WT or YAP-KD L02 cells with or without CytoD treatment were subjected to NanoSight for quantification. *n* = 3. ^∗∗^*p* < 0.01, ns: *p* > 0.05, *t*-test.

To explore the effect of YAP on EV secretion from hepatocytes, we first found that lower YAP expression is correlated with smaller amount of secreted EVs from nanoparticle tracking analysis (NTA) and western blot analysis, by comparing EVs from WT and YAP knockdown (YAP-KD) L02 cells (the normal human hepatocyte line) during hypoxia/reoxygenation (Figure 2(a), Supplementary Fig. 2C, and Figure 2(b)). To elucidate the underlying mechanism of EV secretion in hepatocytes, we explored the role of the dynamic actin and branched actin networks in EV secretion under YAP regulation in vitro, which are on the surface of early endosome (EE). These networks are very important for membrane remodeling, which are critical for selective cargo sorting and EE shape control [7]. We found that most of the WT EE (marked by EEA1) were intensively covered by branched actin networks. In YAP-KD L02 cells, the amount of branched actin on EE decreased significantly (Figure 2(c)). Further analysis of YAP-KD L02 cells revealed that the recycling endosome (RE), which was formed by buddings from EE tubules, also decreased significantly as branched actin formation reduced, indicated by the decreased coposition of EE and F-actin on the plasma membrane (yellow). The RE is the main source of endosomal membrane formation. To study the role of F-actin in EV secretion under the regulation by YAP, we used cytochalasin D (CytoD) to induce F-actin depolymerization for 30 minutes. The depolymerized F-actin restored the inhibition of RE under YAP-KD condition, presenting as an increased level of copositioning of EE and F-actin on the plasma membrane (Figures 2(c) and 2(d)). At the same time, CytoD treatment also reversed the inhibitory effect of YAP-KD on EV secretion (Figure 2(e)). These results suggested that YAP might regulate the formation of EVs via F-actin in hepatocytes.

### 3.3. YAP Expression Is Essential for Inhibiting the Fusion of MVB and Lysosomes

**Figure 3:**
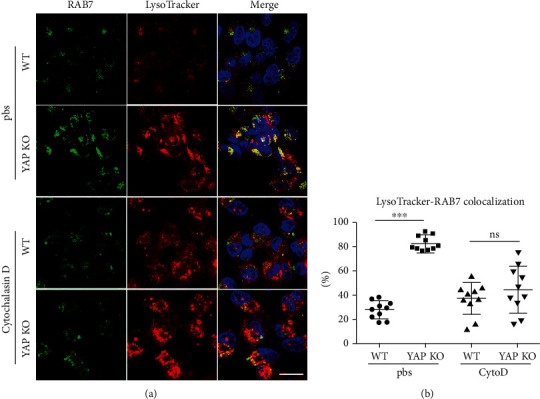
YAP expression is required for the fusion of MVB and lysosomes. (a) Representative image of the fusion of lysosome (labeled by LysoTracker staining, red) with late endosomes (labeled by RAB7 staining, green) significantly increased in YAP-KD compared to WT L02 cells with or without CytoD treatment. Scale bar, 10 *μ*m. (b) Statistical analysis of the amounts of RAB7^+^ granules colocalize (orange) with lysosome, *n* = 10, ^∗∗∗^*p* < 0.001, ns: *p* > 0.05, *t*-test.

Next, we evaluated whether YAP could induce EV secretion by inhibiting MVB and lysosome degradation. The dense reticulum of F-actin might form organelle traps in cells and slow down lysosome transport through an active F-actin anchoring mechanism [31]. We used late endosomes (LE, labeled with RAB7) to indicate MVE. With increased formation of F-actin patches in YAP-KD (Figure 2(c)), the fusion of lysosome (labeled by LysoTracker Red) with LE significantly increased (Figures 3(a) and 3(b)). When F-actin depolymerization was induced by CytoD, there was no significant difference in the colocalization of LE and lysosomal fluorescence between YAP-KD and WT L02 cells (Figure 3(b)). These results suggested that YAP induced EV secretion through F-actin by increasing membrane formation, as well as inhibiting the fusion of MVB and lysosomes.

### 3.4. CD47-Enriched EV Inhibits CD172^+^ DC Activation in a YAP-Dependent Manner

**Figure 4:**
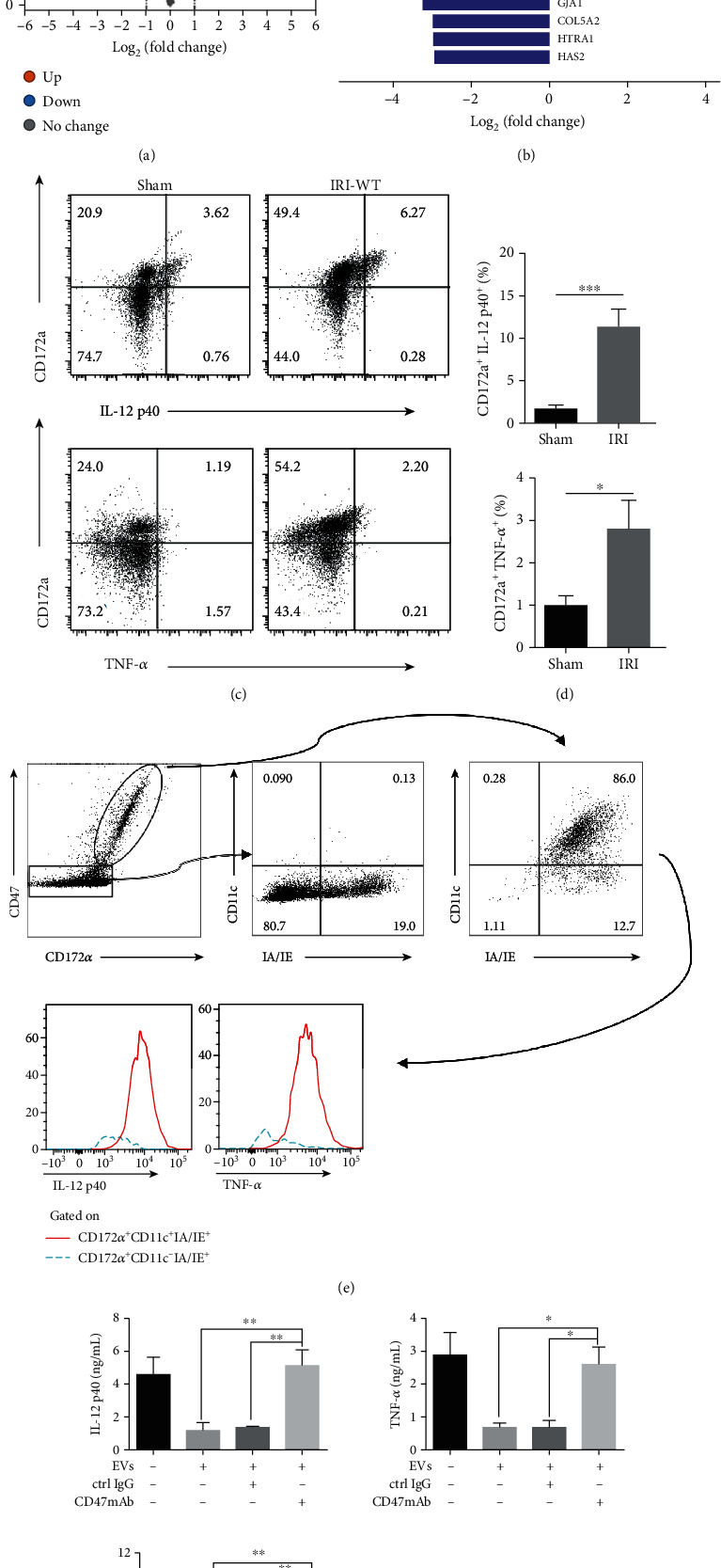
CD47-enriched EVs inhibit CD172a^+^ DC activation. (a) Volcano map presenting the 62 downregulated and 277 upregulated proteins in EVs originated from WT L02 and YAP-KD L02 cells by mass spectrometry, in which CD47 were among the upregulated proteins. (b) Bar chart showing the top 10 upregulated and downregulated proteins with CD47 ranked top four. Protein expression (Ex) was converted to log2 (Ex), and the scale represented relative expression [20]. (c) Representative flow cytometry images of CD45^+^ cells stained by CD172a combined with IL-12 p40 or TNF-*α* in sham or IRI mouse models. (d) The proportion of CD172a^+^ IL-12 p40^+^ or CD172a^+^ TNF-*α*^+^ cells was shown. Bars represent the subset amounts of CD172a^+^ IL-12 p40^+^ or CD172a^+^ TNF-*α*^+^ cells (mean ± s.d., *n* = 4 mice). (e) CD47 and CD172*α* expressions were analyzed on hepatic immune cell populations. CD172*α*^+^ cells were further subdivided according to CD11c and IA/IE expressions. Intracellular expression of cytokines (IL-12 p40 and TNF-*α*) was examined on CD172*α*^+^ CD11c^+^ (solid red lines) and CD172*α*^+^ CD11c^−^ (dotted blue lines) gated cells. (f) PMH-derived EVs were treated by CD47 neutralizing antibody or control IgG. qPCR analysis showed a significant increase in inflammatory cytokines including IL-12 p40, TNF-*α*, and IL-6 after CD47 neutralization. *n* = 6, ^∗^*p* < 0.05; ^∗∗^*p* < 0.01; ^∗∗∗^*p* < 0.001.

To determine the potential effect of YAP-induced EVs on hepatic IRI, we used quantitative mass spectrometry to detect EVs originated from WT L02 and YAP-KD L02 cells. 2883 trusted proteins were retrieved from the original data of mass spectrometry by Protein Pilot software (Supplementary Table 2, Figures 4(a) and 4(b)). Based on this result, we noticed CD47 was one of the target that highly expressed on hepatocyte-derived EVs in a YAP-dependent manner and then verified its expression on primary mouse hepatocytes (PMH) by western blotting (Supplementary Fig. 2D) and immunogold electron microscopy (Supplementary Fig. 2E). CD47 has been reported to exert its inhibitory effect by binding specifically to the surface receptor CD172*α* of myeloid cells [32]. We examined whether CD47-enriched EVs target on the proinflammatory cells during hepatic IRI. By evaluating the inflammatory cytokines IL-12 p40 and TNF-*α* of CD172^+^ cells in hepatic tissue with I/R at 24 h, we found that CD172a^+^ rather than CD172a^−^ cells are the main sources of IL-12 p40 and TNF-*α* (Figure 4(c)). Compared with the sham operation group, the ratio of CD172a^+^ IL-12 p40^+^ or TNF-*α*^+^ cells in the IRI group significantly increased (Figure 4(d)). Furthermore, we found that CD11c expression in hepatic tissues with I/R was limited to the CD172a^+^ cell subset (Figure 4(e)). We next confirmed that among CD172^+^ cells, CD172^+^ CD11c^+^ IA/IE^+^ (MHC II^+^) cells or CD172^+^ DCs represented as the main producers of IL-12 p40 and TNF-*α* in hepatic IRI. These data suggested that the inflammatory cytokine production is restricted to CD172a^+^ DCs in hepatic IRI. As CD47 is located on the surface of EVs, we used CD47 neutralizing antibodies to neutralize EVs and found that neutralizing of CD47 from PMH-EVs significantly antagonized the ability of these EVs to inhibit the secretion of inflammatory cytokines by bone-marrow-derived dendritic cells (BMDCs) (Figure 4(f)). These results suggested that CD47-enriched EVs could suppress inflammatory responses of CD172^+^ DCs.

**Figure 5:**
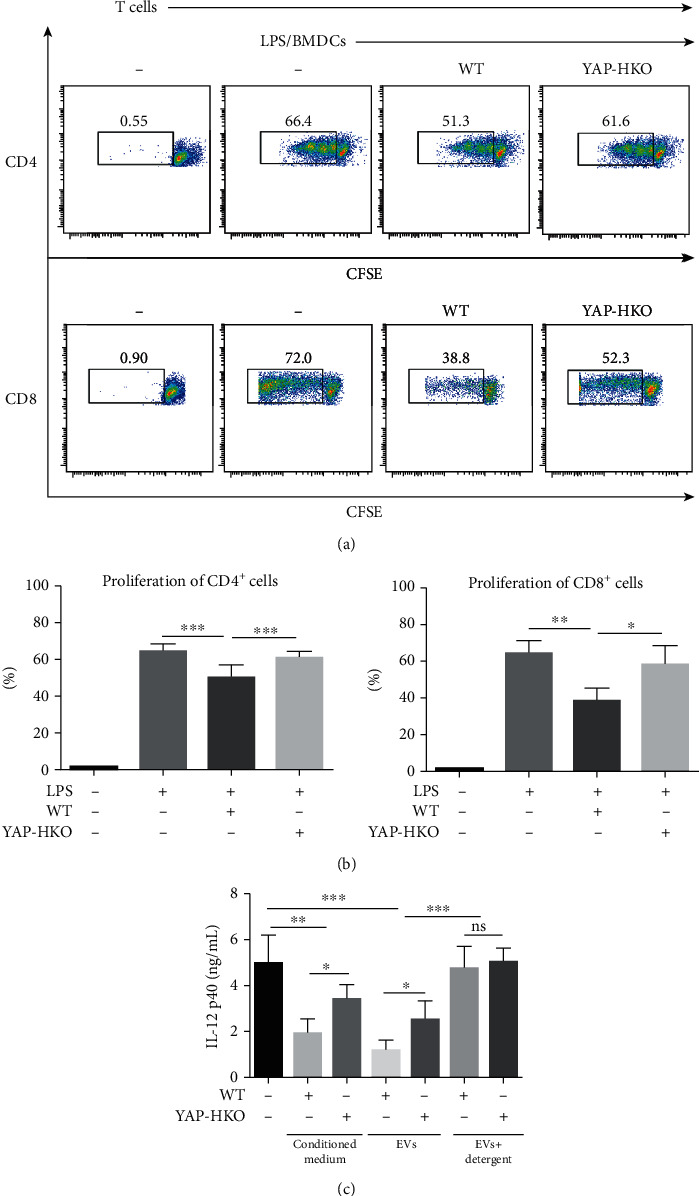
EVs released from hepatocytes inhibit DC activation in a YAP-dependent manner. (a) Primary BMDCs were treated by a conditioned medium from WT or YAP-HKO PMH and were stimulated with LPS. BMDCs were then cocultured with CD4^+^ and CD8^+^ T cells isolated from BALB/c mice at the ratio of 1 : 10. The proliferation of CD4^+^ and CD8^+^ T cells was measured by CFSE-MLR. (b) The extent of T cell proliferation in (a) was analyzed. (c) BMDCs were treated with a conditioned medium or EVs from WT or YAP-HKO PMH culture supernatants, and then, the IL-12 p40 level in the culture medium was determined by ELISA. ^∗^*p* < 0.05; ^∗∗^*p* < 0.01; ^∗∗∗^*p* < 0.001. *n* = 4.

To evaluate the antigen-presenting function of DCs, we detected the proliferation of CD4^+^ and CD8^+^ T cells in vitro. In comparison to the YAP-HKO PMH treatment group, we found the proliferation rates were inhibited in BMDCs pretreated by a conditioned medium of WT PMH (Figures 5(a) and 5(b)). Meanwhile, by detecting IL-12 p40, we found LPS-induced-BMDC activation was significantly restrained by a PMH conditioned medium in a YAP-dependent manner (Figure 5(c)). These results implied that, in the absence of YAP, hepatocytes lose their ability to suppress BMDC activation in vitro.

To elucidate that YAP-dependent-DC function was regulated by hepatocyte-released EVs, we isolated EVs by ultracentrifugation from the supernatant of PMH. We found EVs isolated from WT were more potent in inhibiting BMDCs than EVs from YAP-HKO PMH, as evaluated by IL-12 p40 secretion (Figure 5(c)). To distinguish between EVs and other nonmembrane extracellular particles [20], EVs were treated with detergent (Triton X-100) which can destroy the membrane structure and halt the functions of EVs. The particles after detergent treatment failed to inactivate BMDCs (Figure 5(c)). These results indicated that DC activation was inhibited by hepatocyte-released EVs in a YAP-dependent manner.

### 3.5. CD47^+^ EVs Could Alleviate Hepatic IRI as a Therapeutic Strategy

**Figure 6:**
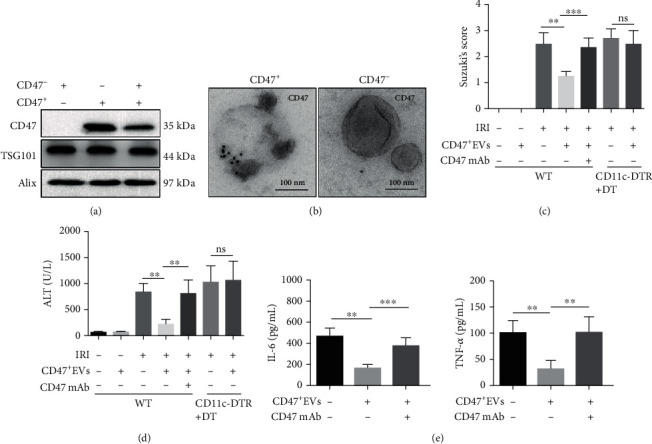
CD47^+^ EVs alleviate hepatic IRI in mouse models. (a) CD47^+^ EVs and CD47^−^ EVs were isolated by nonblocking anti-CD47 Ab (REA170)-FITC and anti-FITC magnetic beads. Alix, TSG101, and CD47 in CD47^+^, CD47^−^, and total PMH-EVs were checked by western blot. (b) Immunogold electron microscopy images showed the immunoreactivity for CD47 on CD47^+^ and CD47^−^ EVs. (c) CD11c-DTR were treated with DT (4 mg/kg b.w.) and/or plus with CD47^+^ EVs (100 *μ*g/kg b.w.) on 2 and 0 days before IRI model construction. The severity of hepatic IRI was evaluated by Suzuki's grading [33]. (d) Hepatocellular function was assessed by serum ALT level (U/L). (e) ELISA analysis showed decreased concentrations of IL-6 and TNF-*α* after CD47^+^ EVs supply without CD47mAb treatment during IRI. *n* = 6, ^∗^*p* < 0.05; ^∗∗^*p* < 0.01; ^∗∗∗^*p* < 0.001.

To investigate whether CD47^+^ EVs have the potential to treat hepatic IRI, we first isolated CD47^+^ EVs and CD47^−^ EVs using nonblocking anti-CD47 Ab (REA170)-FITC and anti-FITC magnetic beads. We then verified the purified CD47^+^ EVs contain CD47 and EVs' label proteins TSG101 and Alix (Figures 6(a) and 6(b)). Next, we injected CD47^+^ EVs into WT mice to determine whether CD47^+^ EVs have any adverse effect, and we found there was no damage to normal mice without IRI. While in the IRI model, less hepatic damage was observed in the group treated by CD47^+^ EVs (Figures 6(c) and 6(d)). We found the expression of antioxidative genes increased including thioredoxin-1 (Trx1) and heme oxygenase-1 (HO-1) after CD47^+^ EV treatment. Further, we examined the expression of Cyr61, which has been recognized as a key chemokine controlling liver injury [5]. The mRNA levels of hepatic Cyr61 were reduced after CD47^+^ EV treatment (Supplementary Fig. 2F). As CD47mAb could reverse the protective effects of CD47^+^ EVs, the protection of these EVs on hepatic IRI is possibly mediated by CD47 (Figures 6(c) and 6(d)). In addition, there was no significant amelioration in hepatic damage after I/R in CD11c-DTR mice injected with DT after CD47^+^ EV treatment (Figures 6(c) and 6(d)), suggesting that CD47^+^ EVs alleviate hepatic IRI at least partially depending on DCs. Similarly, CD47^+^ EV injection reduced the serum levels of IL-6 and TNF-*α*, while this effect was reversed by CD47mAb (Figure 6(e)). These results showed that CD47^+^ EVs may be a potential therapeutic strategy for the treatment of hepatic IRI.

Taken together, our data showed that YAP regulates the secretion of hepatocyte-derived EVs, which can inhibit DC activation through its surface CD47 and contribute to liver protection during hepatic IRI.

## 4. Discussion

In this study, we identified a novel mechanism that YAP activation affects the release of hepatic EVs, which regulates DC activation through CD47/CD172a axis. CD47-enriched EVs could be a novel therapeutic strategy for treating hepatic IRI by targeting on CD172a^+^ DCs.

The participation of YAP in IRI has been reported in different studies with controversial effects [4, 5]. In our study, hepatic YAP protein and mRNA were induced in a time-dependent manner in hepatic IRI, demonstrating the requisite role of YAP. This notion was strongly supported by human OLT samples. Further, we reported that conditional knockout YAP in hepatocytes led to severe hepatic damage with IRI, by crossing Albumin-Cre (*Alb-Cre*) mice and *Yap^flox/flox^* mice [34]. These mice displayed increased serum ALT levels and pathological hepatic changes, indicating YAP silencing played a pivotal role in aggregating hepatic IRI. Then, via serum EV isolation kit, we found in the serum of YAP-HKO mice, the amount of sEVs decreased after IRI. Thus, we further investigated how YAP knockout affects sEV secretion and exacerbates hepatic injuries.

Actually, it has been found that IRI can induce the secretion of EVs [29], but how YAP knockout in IRI decreased EV secretion remains unclear. EVs are formed through direct germination of plasma membrane or germination of endomembrane structure after fusing with late endosomes (LE) or multivesicular body (MVB) and are secreted based on the process that fuses with plasma membrane [35]. During this process, the branched actin network is known to play a key role in endosomal trafficking and EV secretion [36]. YAP can regulate the actin-binding activity of AMOT family members by competing with F-actin for binding to AMOT130 [6]. It is possible that YAP may regulate EV secretion by regulating F-actin. In our study, we investigated the mechanism involved in endosomal trafficking, which involves the Rab family of small GTPases, lysosomes, and remodeled actin [37]. We found that YAP knockdown by lentivirus shRNA significantly reduced EV release, suggesting that YAP participated in EV secretion. By comparing WT and YAP-KD hepatocytes, we found that YAP-KD decreased the formation of branching actin. By limiting the fusion of intraluminal vesicles and cell membranes, it is conducive to the development of RE and further decreases the occurrence of EV membrane [24, 38]. RE is closely related to the generation of EVs [39]. Both dynamic and branched actin could reduce the fusion of LE and lysosome, thereby increasing the secretory MVE as EVs. Therefore, we are the first to report that YAP from hepatocytes regulate EV secretion through F-actin in hepatic IRI. But our works focused on the role of EVs released from hepatocytes; further studies are needed to explore the effect of EVs from other nonparenchymal cells, such as Kupffer cells, liver sinusoidal endothelial cells, and immune cells. Besides, autophagy has been reported to promote the fusion of MVB and autophagy [40], and YAP is well known to regulate autophagy flux by promoting autolysosome degradation [41]. Whether YAP regulated EV secretion through autophagy requires further study.

Then, to investigate the potential effect and mechanism of EVs in hepatic IRI, we applied mass spectrometry to explore the essential elements. Among the top factors, we found that CD47 exists on the surface of EV membrane. CD47 is considered to be the self-recognition marker that specially binds CD172*α*, which profoundly inhibits the secretion of inflammatory cytokines. The latest tumor research uses CD47 to modify nanoparticles, which reduces the clearance rate of nanoparticles by macrophages [42]. Studies have shown that CD47 not only takes part in autoimmune diseases and tumors but also plays a key role in IRI-related diseases in kidneys and hearts [43, 44]. And CD172*α*^+^ DCs, as target cells of CD47, are the main cells secreting proinflammatory cytokines in hepatic IRI [15]. CD47mAb blockade confirmed that EV-associated CD47 take the immunosuppressive effect on DCs and reduced hepatic IRI. Under normal conditions, DCs remain in an inactivated state. Under inflammation stress, DCs are exposed to foreign antigens, microorganisms, and inflammatory cytokines resulting in activation and then subsequently initiating immune response [28]. Our results highlighted the regulatory role of YAP in the regulation of DC function during hepatic IRI. In this study, we observed that hepatocyte culture medium obviously inhibited LPS-induced IL-12 p40 production and antigen-presenting ability of BMDCs in a YAP-dependent manner. We suspected that under stress, YAP induced EV secretion from hepatocytes, which have the immunosuppressive effect on DCs. Interestingly, we treated EVs with detergent, which destructed the membrane structures of EVs, reversing the inhibitory effect of EVs on DCs. This suggested that YAP induced the secretion of EVs to exert an immunosuppressive effect on DCs. In addition, we found that CD47+ EVs can inhibit DC activation and inflammatory responses, which can explain their protective effects on IRI. Together, these data suggest that CD47-enriched-EVs can inhibit DC activation and decrease subsequent hepatic damage.

Innate immunity is the dominant ingredient of liver IRI, among which dendritic cells, macrophage, and neutrophils are pivotal participants [45]. In comparison with neutrophils and macrophages, DCs are the dominant local immune cells surveilling and maintaining immune homeostasis with less complex phenotype switches [46]. Moreover, DC has been determined as the main contributors in hepatic IRI, affecting the development of adaptive immunity by regulating the initial response and amplifying the innate immune response [11]. The role of other immune cells such as macrophages or neutrophils in hepatic IRI needs further investigation.

EVs have been proved to be ideal cargos for drug delivery, which showed better stability, easier preservation, and more precise targeting in comparison to traditional cell therapy or RNA drugs [47]. The advantages of EVs make them attractive as the potential candidates for treating many diseases. The specific structure of phospholipid bilayer enables EVs to protect their contents from in vivo degradation and the disturbances from inflammatory microenvironment [48]. Besides, EVs could be modified and produced in a standard process. Therefore, CD47-enriched EVs could be engineered by integrating EVs with CD47 loading, which could be manufactured at a large scale independent of the cell origin, which could be the future direction of precise drug development.

**Figure 7:**
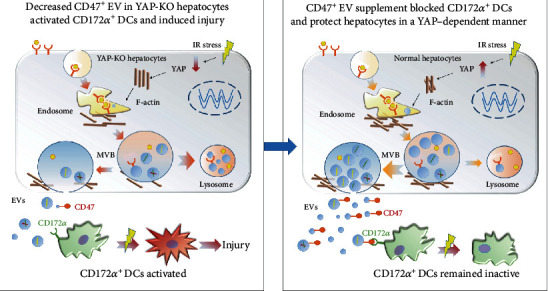
Mechanism diagram for the immunosuppression effect of YAP/F-actin/EV-CD47 axis on DCs in hepatic IRI. IR stress stimulated YAP expression in normal hepatocytes and promoted the remodeling of F-actin to form an endosomal trafficking network, which is critical for EV formation. These EVs were rich in CD47, which could bind to CD172a receptor on DCs. In normal conditions, most of the MVB would form into EVs with a little few into lysosome. When YAP expression was knocked down, however, F-actin failed to gather around and form a network report; thus, the amount of EVs decreased with more vesicles engulfed by lysosome. The amount of CD47 was scarce, and CD172^+^ DCs were activated and led to more severe damage. Therefore, EVs lack of CD47^+^ due to YAP knockout in hepatocytes, activated DCs, and aggregated hepatic IRI, while YAP activation induced by hepatic IRI promoted the secretion of CD47-enriched EVs by remodeling of cytoskeleton F-actin, and CD47^+^ EVs induced the immunosuppressive DCs and protected the liver from IRI. CD47^+^ EV supplement could be a novel therapeutic strategy for hepatic IRI treatment.

In summary, our study reported a novel regulatory mechanism between EVs and DCs depending on YAP activation in hepatic IRI (Figure 7). Specifically, YAP knockout decreased the release of EVs in hepatocytes by affecting F-actin, which reduced membrane formation and promoted MVB to fuse into lysosomes. The YAP-deficient EVs are lack of CD47, which failed to inactivate CD172*α*^+^ DCs and lead to sustained IRI injury. CD47-enriched EV treatment blocked CD172*α*^+^ DCs and protected IRI mice from hepatic injury. Our findings suggested that the secretion of CD47^+^ EVs depending on YAP activation played a protective role during hepatic IRI, and CD47-enriched EVs could be a novel therapeutic strategy for hepatic IRI treatment.

## Data Availability

All data relevant to the study are included in the article or uploaded as supporting information.

## Abbreviations

ALT: Alanine aminotransferase
AMOT: Angiomotin
BM: Bone marrow
CytoD: Cytochalasin D
DCs: Dendritic cells
DT: Diphtheria toxin
EVs: Extracellular vesicles
HO-1: Heme oxygenase-1
I/R: Ischemia-reperfusion
IRI: Ischemia-reperfusion injury
LPS: Lipopolysaccharide
MVB: Multivesicular body
MLR: Mixed leukocyte reaction
NanoSight: Nanoparticle tracking analysis
OLT: Orthotopic liver transplantation
PMH: Primary mouse hepatocyte
SIRPa: Signal-regulated protein alpha
Trx1: Thioredoxin-1
WT: Wild-type
YAP: Yes-associated protein
YAP-HKO: Hepatocyte-specific YAP knockout.

## Supplementary Materials

Supplementary Materials Supplementary Figure 1: (A) representative images of YAP expression in liver graft by immunohistochemistry (left panel), 37 patients were in the YAP high-expression group and 32 patients were in the YAP low-expression group; representative histology of liver by H&E staining (right panel) from indicated groups. Magnification ×200. (B) Suzuki's histological grading of the YAP high-expression group was significantly lower than the low-expression group (score: 1.60 ± 0.08 vs. score: 2.37 ± 0.13, ^∗∗∗^*p* < 0.001). (C) The peak serum ALT within 7 days after transplantation of the high-expression group were significantly lower than the low-expression group (448.4 ± 22.78 U/L vs. 653.9 ± 36.44 U/L, ^∗∗∗^*p* < 0.001). (D) The schematic diagram of design strategy of YAP-HKO mice, and the plasmid map of the targeting vector used in YAP-HKO mice. YAP-HKO mice were constructed by crossing Albumin-Cre (*Alb-Cre*) mice and *Yap^flox/flox^* mice. (E) YAP protein levels of the liver from two independent WT and two independent YAP-HKO mice were measured by western blot. Supplementary Figure 2: (A) EVs isolated from mouse serum were visually confirmed by transmission electron microscopy, and (B) subjected to western blot analysis with antibodies to the indicated proteins. (C) WT L02 cells secrete more EVs. EVs isolated from culture supernatants of WT or YAP knockout (KO) L02 cells were subjected to nanoparticle tracking analysis (NanoSight) to quantify the number and size distribution. (D) A total of 35 *μ*g of EVs isolated from culture supernatants of WT or YAP-HKO PMH were analyzed by western blot using the indicated antibodies. Data are representative of three independent experiments. (E) Immunogold electron microscopy analysis depicts the presence of CD47 on the surface of PMH-derived EVs. (F) Quantitative RT-PCR-assisted detection of Trx1, HO-1, and Cyr61 after CD47^+^ EV treatment during IRI. Supplementary Table 1: demographics, perioperative situation, and laboratory results of liver transplantation surgery patients. Supplementary Table 2: the 62 downregulated and 277 upregulated proteins in EVs originated from WT L02 and YAP-KD L02 cells by mass spectrometry.
