# Supplementary material for: YAP-Dependent Induction of CD47-Enriched Extracellular Vesicles Inhibits Dendritic Cell Activation and Ameliorates Hepatic Ischemia-Reperfusion Injury
Source: Oxid Med Cell Longev. 2021 Jun 22;2021:6617345. doi: 10.1155/2021/6617345 (PMC8241504; doi:10.1155/2021/6617345)
Supplement: Supplementary Materials — Supplementary Figure 1: (A) representative images of YAP expression in liver graft by immunohistochemistry (left panel), 37 patients were in the YAP high-expression group and 32 patients were in the YAP low-expression group; representative histology of liver by H&E staining (right panel) from indicated groups. Magnification ×200. (B) Suzuki's histological grading of the YAP high-expression group was significantly lower than the low-expression group (score: 1.60 ± 0.08 vs. score: 2.37 ± 0.13, ∗∗∗p < 0.001). (C) The peak serum ALT within 7 days after transplantation of the high-expression group were significantly lower than the low-expression group (448.4 ± 22.78 U/L vs. 653.9 ± 36.44 U/L, ∗∗∗p < 0.001). (D) The schematic diagram of design strategy of YAP-HKO mice, and the plasmid map of the targeting vector used in YAP-HKO mice. YAP-HKO mice were constructed by crossing Albumin-Cre (Alb-Cre) mice and Yapflox/flox mice. (E) YAP protein levels of the liver from two independent WT and two independent YAP-HKO mice were measured by western blot. Supplementary Figure 2: (A) EVs isolated from mouse serum were visually confirmed by transmission electron microscopy, and (B) subjected to western blot analysis with antibodies to the indicated proteins. (C) WT L02 cells secrete more EVs. EVs isolated from culture supernatants of WT or YAP knockout (KO) L02 cells were subjected to nanoparticle tracking analysis (NanoSight) to quantify the number and size distribution. (D) A total of 35 μg of EVs isolated from culture supernatants of WT or YAP-HKO PMH were analyzed by western blot using the indicated antibodies. Data are representative of three independent experiments. (E) Immunogold electron microscopy analysis depicts the presence of CD47 on the surface of PMH-derived EVs. (F) Quantitative RT-PCR-assisted detection of Trx1, HO-1, and Cyr61 after CD47+ EV treatment during IRI. Supplementary Table 1: demographics, perioperative situation, and laboratory results of liver transplant [file 6617345.f1.pdf]

Supplementary Materials and Methods

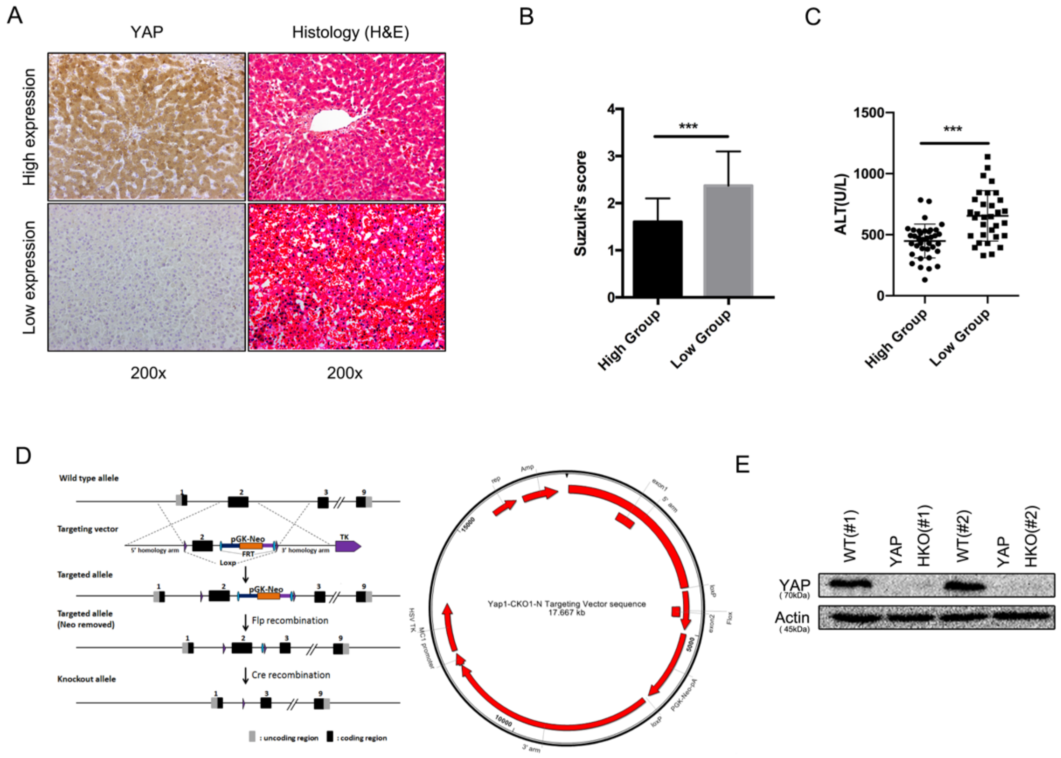

Supplementary Fig 1. (A) Representative images of YAP expression in liver graft by immunohistochemistry (left panel), 37 patients were in YAP high-expression group and 32 patients were in YAP low-expression group; representative histology of liver by H&E staining (right panel) from indicated groups. Magnification x200. (B) The Suzuki's histological grading of YAP high-expression group was significantly lower than low-expression group (score: 1.60 ± 0.08 vs. score: 2.37 ± 0.13, *** $p < 0.001$). (C) The peak Serum ALT within 7 days after transplantation of high- expression group were significantly lower than low-expression group (448.4 ± 22.78 U/L vs. 653.9 ± 36.44 U/L, ***$p<0.001$). (D) The schematic diagram of design strategy of YAP-HKO mice, and the plasmid map of the targeting vector used in YAP-HKO mice. YAP-HKO mice were constructed by crossing Albumin-Cre (*Alb-Cre*) mice and *Yap*$^{flox/flox}$ mice. (E) YAP protein levels of liver from two independent WT and two independent YAP-HKO mice were measured by Western blot.

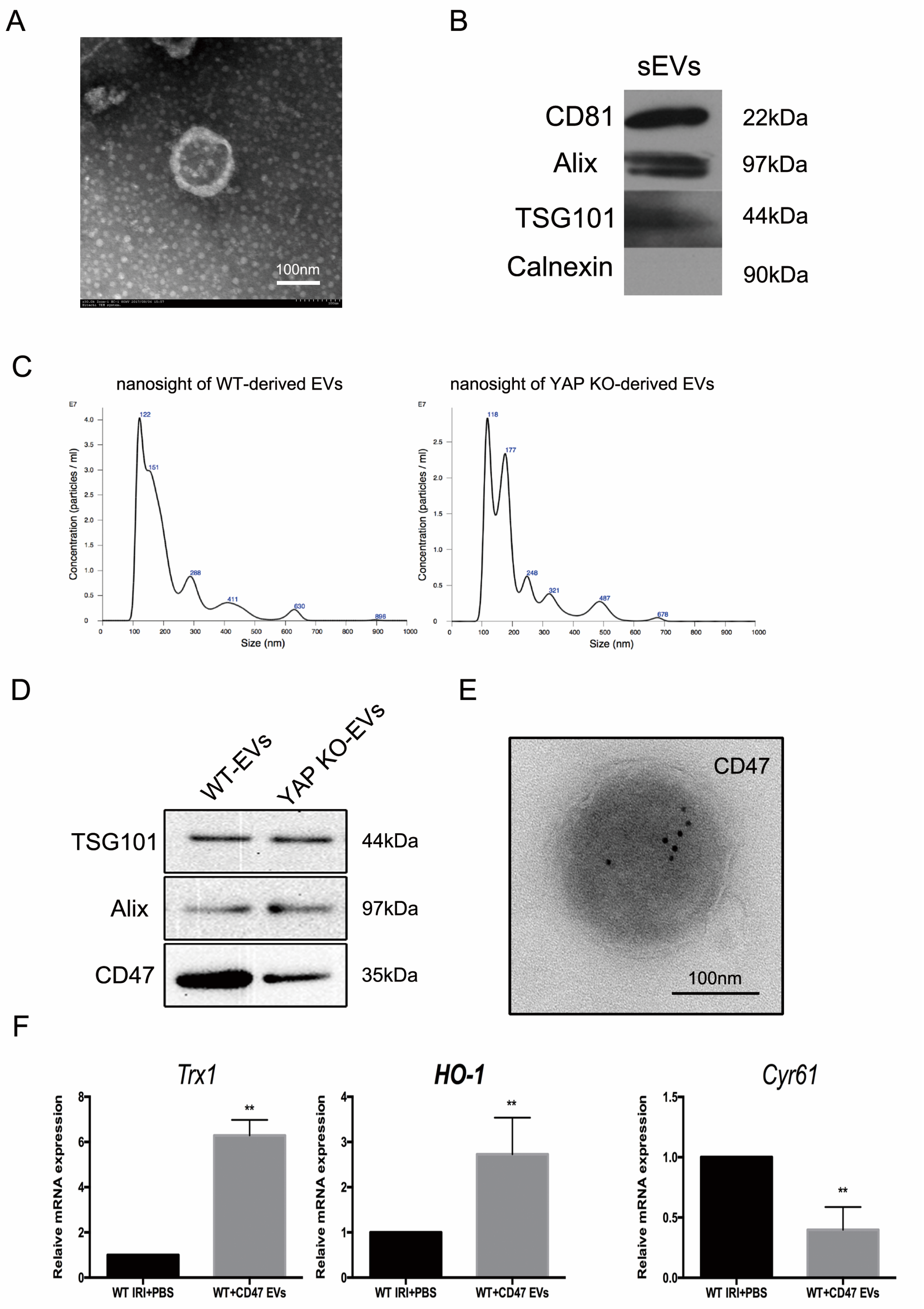

Supplementary Fig 2. (A) EVs isolated from mice serum were visually confirmed by transmission electron microscopy, (B) and subjected to western blot analysis with antibodies to the indicated proteins. (C) WT L02 cells secrete more EVs. EVs isolated from culture supernatants of WT or YAP knockout (KO)

L02 cells were subjected to nanoparticle tracking analysis (NanoSight) to quantify the number and size distribution. (D) A total of 35 μg of EVs isolated from culture supernatants of WT or YAP-HKO PMH were analysed by western blot using the indicated antibodies. Data are representative of three independent experiments. (E) immunogold electron microscopy analysis depicts presence of CD47 on surface of PMH-derived EVs. (F) Quantitative RT-PCR-assisted detection of Trx1, HO-1 and Cyr61 after CD47+ EVs treatment during IRI.

Supplementary Table 1 Demographics, perioperative situation and laboratory results of liver transplantation surgery patients

numbers	sex	age	Preoperative liver function grade	cold ischemia time（h）	non-hepatic period（min）	Degree of IRI	score of YAP
383776	male	67	2	4	55	mild	0
387929	male	36	1	5	65	mild	0
355487	male	58	1	4	39	mild	0
908323	male	41	2	6	38	mild	0
381310	female	59	3	4	100	moderate	2
906903	male	48	2	5	45	moderate	1
906255	male	47	1	4	25	moderate	3
369381	male	58	2	6	35	mild	2
268662	male	60	3	7	42	mild	4
271681	male	35	2	7	42	moderate	1
224556	male	46	1	6	45	moderate	0
261556	male	31	2	6	45	moderate	3
270306	male	40	2	3	40	mild	0
267676	male	44	1	6	40	mild	6
268515	male	47	1	6	40	mild	6
265011	male	56	3	5	45	moderate	0
262695	male	52	1	5	30	moderate	2
262504	male	48	1	5	45	moderate	0
258541	male	40	1	3	40	mild	6
257896	male	45	1	5	45	moderate	2
256651	male	49	3	8	60	moderate	0
254738	male	55	1	5	45	moderate	1
249799	male	24	2	5	40	moderate	0
244526	male	37	1	10	40	moderate	4
239625	male	46	1	8	40	mild	0
239561	female	21	1	11.5	50	moderate	2

237983	male	45	3	11	35	moderate	6
234714	male	29	1	4	40	moderate	1
229804	male	36	3	8	40	mild	1
226681	male	46	1	11	60	moderate	0
220295	male	39	1	12	30	mild	0
219195	male	49	2	9	46	moderate	0
218932	male	37	1	3	35	moderate	2
218403	male	55	2	10	65	mild	0
906567	male	32	1	8	45	moderate	2
340680	male	52	1	4	32	mild	0
333625	male	32	2	6	28	mild	1
322555	male	57	1	5	40	mild	0
903446	male	58	2	8	50	moderate	2
352968	male	48	3	6	50	mild	0
367100	male	57	3	6	36	mild	0
356182	male	50	3	2		mild	0
322850	male	56	2	6	53	mild	0
902000	male	59	2	6	35	mild	0
902237	male	30	2	6	35	mild	0
268584	male	63	1	6	45	mild	0
269104	male	35	2	6	45	moderate	0
269191	male	65	2	5	35	mild	0
267563	female	53	1	3	35	mild	4
157685	female	68	1	8	27	mild	1
262873	male	47	1	6	44	mild	4
251722	male	54	1	6	35	mild	0
260175	male	53	1	3	35	mild	2
259745	male	40	1	5	28	mild	0
257812	male	47	1	6	45	mild	0
227541	male	43	1	4	55	mild	0
252289	male	64	3	5	63	moderate	0
251846	male	61	1	3	45	mild	4
251830	male	34		5	40	mild	9
244546	male	54	1	10	45	moderate	0
243246	male	51		6	40	mild	0
132566	female	37	1	2	97	moderate	12
241970	male	37		6	40	mild	0
237986	male	55	1	13.5	55	moderate	2
238341	male	72	3	8	45	moderate	1
235350	male	36	3	9	35	mild	2
235819	male	35	1	10	75	moderate	0
183069	male	44	3	6	35	mild	0
235345	male	68	1	4	40	mild	0

Supplementary Table 2 the 62 downregulated and 277 upregulated proteins in EVs originated from WT L02 and YAP-KD L02 cells by mass spectrometry

N	Citable Accession	Gene Name	C.V	AVG.	T.TEST	FLAG
1	Q09666	GN=AHNAK	0.28015923	2.731924206	0.029537764	UP
2	P02751	GN=FN1	0.290769207	0.091126614	1.05055E-05	DOWN
4	P21333	GN=FLNA	0.343701677	2.806990236	0.047702445	UP
5	P49327	GN=FASN	0.2697075	5.769987106	0.013048938	UP
6	Q14204	GN=DYNC1H1	0.123661	1.687799007	0.010666985	UP
9	P46940	GN=IQGAP1	0.197803871	4.089984477	0.007033484	UP
10	O15230	GN=LAMA5	0.292824345	2.602692753	0.035683121	UP
12	P02452	GN=COL1A1	0.443208845	0.076216953	2.07187E-05	DOWN
15	P18206	GN=VCL	0.218789085	3.878288686	0.009836666	UP
16	O75369	GN=FLNB	0.320103105	2.956156254	0.037272471	UP
18	P14618	GN=PKM	0.110652495	4.184396207	0.001272272	UP
20	P31327	GN=CPS1	0.045584813	1.755703986	0.000497432	UP
23	Q14764	GN=MVP	0.175782588	1.572991729	0.037042134	UP
24	P11142	GN=HSPA8	0.050762138	0.525500655	7.51237E-05	DOWN
27	P07900	GN=HSP90AA1	0.356609863	3.329685241	0.042514511	UP
28	P05187	GN=ALPP	0.395342723	5.019846439	0.039243721	UP
32	O43707	GN=ACTN4	0.120792443	2.839023232	0.002641325	UP
33	O00468	GN=AGRN	0.220702822	2.65999198	0.016289632	UP
35	P26038	GN=MSN	0.176291966	2.611210048	0.00900678	UP
36	P08133	GN=ANXA6	0.238919357	2.097123027	0.032165373	UP
37	O00159	GN=MYO1C	0.279470825	2.149544775	0.045243367	UP
38	P55268	GN=LAMB2	0.196033871	3.044153988	0.009570125	UP
39	P06733	GN=ENO1	0.181067707	3.176756084	0.007220869	UP
40	Q14315	GN=FLNC	0.249790873	2.521325052	0.024884374	UP
41	P78371	GN=CCT2	0.190309237	1.793902725	0.027504671	UP
42	P33527	GN=ABCC1	0.194066475	2.114240736	0.018182596	UP
44	P02786	GN=TFRC	0.097434662	2.333608449	0.002032276	UP
45	P08195	GN=SLC3A2	0.17018544	3.344879031	0.005668191	UP
48	P08123	GN=COL1A2	0.411300419	0.083279999	2.2103E-05	DOWN
49	P12111	GN=COL6A3	0.409176523	0.148973091	0.000155006	DOWN
50	P04075	GN=ALDOA	0.064548431	2.505570233	0.000518897	UP
51	P13639	GN=EEF2	0.195026924	3.526557982	0.007856096	UP
54	P22314	GN=UBA1	0.220473274	3.053213835	0.013227072	UP
55	Q08380	GN=LGALS3BP	0.17740522	9.504686594	0.00315799	UP
57	Q71U36	GN=TUBA1A	0.235843275	1.798728704	0.047090638	UP
60	P29401	GN=TKT	0.245595006	2.712415487	0.021083905	UP
62	P04406	GN=GAPDH	0.122592627	5.361161709	0.001413972	UP

64	P11413	GN=G6PD	0.286602618	4.319261611	0.018819604	UP
66	P20020	GN=ATP2B1	0.246216232	2.007104754	0.038645105	UP
67	P07814	GN=EPRS	0.0826361	2.23599121	0.001380818	UP
70	P05997	GN=COL5A2	0.303035028	0.125275521	3.46156E-05	DOWN
72	Q08211	GN=DHX9	0.184805453	2.418171704	0.011850462	UP
75	P49588	GN=AARS	0.162847697	3.620951712	0.004554656	UP
76	P00558	GN=PGK1	0.377344268	3.932203233	0.041757419	UP
77	P53621	GN=COPA	0.081949363	1.586591512	0.004362988	UP
78	P05556	GN=ITGB1	0.213406622	7.030929089	0.006080582	UP
79	P32004	GN=L1CAM	0.191202692	4.386245072	0.006002285	UP
82	P14923	GN=JUP	0.075912423	2.19417128	0.001125361	UP
84	P34932	GN=HSPA4	0.063965927	1.745297253	0.001388939	UP
85	Q14974	GN=KPNB1	0.079842161	2.164576292	0.001351266	UP
86	Q08722	GN=CD47	0.156708167	7.418037415	0.002425973	UP
87	P02461	GN=COL3A1	0.152561947	0.280603025	8.90546E-05	DOWN
88	P20908	GN=COL5A1	0.361121211	0.320441131	0.002024895	DOWN
89	O75643	GN=SNRNP200	0.226322933	2.866931021	0.015531656	UP
91	P30464	GN=HLA-B	0.101448601	1.507386506	0.010465488	UP
93	P80723	GN=BASP1	0.419465142	13.1397686	0.03168177	UP
94	Q9P273	GN=TENM3	0.124276925	2.139781713	0.005057526	UP
96	P06737	GN=PYGL	0.240661061	2.272920489	0.027454456	UP
97	P62258	GN=YWHAE	0.288776876	3.061329007	0.027311621	UP
98	P11586	GN=MTHFD1	0.25732303	3.400964439	0.017686045	UP
99	P23229	GN=ITGA6	0.146710449	2.08293128	0.008697155	UP
100	P49368	GN=CCT3	0.230793501	1.744617462	0.049215143	UP
101	O15031	GN=PLXNB2	0.124020538	1.557264477	0.01541227	UP
104	Q15063	GN=POSTN	0.343199842	0.034336902	7.71186E-07	DOWN
105	Q01813	GN=PFKP	0.134854556	2.055822223	0.00709179	UP
106	P26639	GN=TARS	0.226734923	2.748403996	0.01663885	UP
107	P06744	GN=GPI	0.195880775	2.609074771	0.012113496	UP
108	P08238	GN=HSP90AB1	0.007651107	2.142953038	1.25259E-06	UP
114	P27105	GN=STOM	0.336200104	4.57566154	0.027538541	UP
115	P30041	GN=PRDX6	0.351537294	3.154957324	0.043559373	UP
116	P55786	GN=NPEPPS	0.163682603	1.848261774	0.016668021	UP
117	O75874	GN=IDH1	0.201084267	2.264336258	0.017115285	UP
118	P50995	GN=ANXA11	0.272562996	2.087849468	0.045357127	UP
120	O94813	GN=SLIT2	0.377681854	0.561460376	0.03723384	DOWN
122	P35241	GN=RDX	0.329575364	4.51160866	0.026410537	UP
123	P62937	GN=PPIA	0.230549728	4.176447213	0.010635298	UP
124	O14980	GN=XPO1	0.084949929	2.72011131	0.00100703	UP
125	Q6YHK3	GN=CD109	0.074369453	2.832539737	0.000634595	UP
126	P12814	GN=ACTN1	0.113447678	3.722241223	0.001539604	UP
128	Q16658	GN=FSCN1	0.108096695	1.673210263	0.007568873	UP

129	P56192	GN=MARS	0.078224541	2.724771261	0.000786523	UP
130	P35221	GN=CTNNA1	0.184723644	2.043825299	0.017318329	UP
132	P60174	GN=TPI1	0.17579022	1.844244272	0.02036357	UP
134	P63092	GN=GNAS	0.285662664	3.098692805	0.026140231	UP
135	P37802	GN=TAGLN2	0.231801568	1.870248258	0.040146154	UP
140	P11021	GN=HSPA5	0.160908496	0.519551016	0.002157299	DOWN
141	Q9Y262	GN=EIF3L	0.061376461	1.502516776	0.002520706	UP
146	Q14126	GN=DSG2	0.204802031	2.214628786	0.0188834	UP
148	P63104	GN=YWHAZ	0.148189662	2.487171769	0.006013922	UP
149	O00186	GN=STXBP3	0.223813527	2.248855501	0.023174829	UP
151	P31939	GN=ATIC	0.160666767	1.993735492	0.012620959	UP
152	Q12965	GN=MYO1E	0.215033409	1.978014737	0.02832903	UP
154	Q16555	GN=DPYSL2	0.119408949	2.104340494	0.004705376	UP
157	P00966	GN=ASS1	0.328292659	3.472058177	0.032971406	UP
158	P26641	GN=EEF1G	0.08177115	2.006553471	0.001781366	UP
162	P07195	GN=LDHB	0.349881646	4.042487085	0.033672147	UP
163	Q96AC1	GN=FERMT2	0.199357879	2.176568776	0.018257952	UP
164	P18669	GN=PGAM1	0.15066689	3.890853465	0.003371884	UP
167	P04264	GN=KRT1	0.401102507	0.257435601	0.001115249	DOWN
168	P61224	GN=RAP1B	0.221274853	2.068670541	0.027221746	UP
170	Q96QK1	GN=VPS35	0.013662363	2.371594012	5.59139E-06	UP
171	P16070	GN=CD44	0.190257892	2.984761834	0.00904319	UP
172	P41252	GN=IARS	0.243412042	2.198082298	0.030354369	UP
174	Q9Y6C2	GN=EMILIN1	0.382332015	0.188116748	0.000292306	DOWN
175	P09651	GN=HNRNPA1	0.146346985	1.647004992	0.018763759	UP
177	O43143	GN=DHX15	0.120936441	1.968733281	0.005872061	UP
179	P24821	GN=TNC	0.35587435	0.346022643	0.002717245	DOWN
185	Q9Y678	GN=COPG1	0.094362848	2.103503972	0.002377335	UP
187	P23528	GN=CFL1	0.272080938	4.550213873	0.015675113	UP
190	P34897	GN=SHMT2	0.014562225	1.905662745	1.21961E-05	UP
191	O75083	GN=WDR1	0.179473588	2.167523533	0.013831008	UP
192	P61981	GN=YWHAG	0.221683518	1.896300554	0.034445889	UP
193	P07996	GN=THBS1	0.264451114	0.493544623	0.006723695	DOWN
195	P40227	GN=CCT6A	0.261084059	4.194153011	0.014958969	UP
196	O95782	GN=AP2A1	0.011948773	1.749972016	9.18993E-06	UP
198	P61158	GN=ACTR3	0.039073652	1.586103499	0.000495126	UP
199	P08174	GN=CD55	0.337180484	3.670315027	0.033406653	UP
204	Q02818	GN=NUCB1	0.489870208	0.078203186	3.04019E-05	DOWN
205	P63244	GN=RACK1	0.211810513	1.90435794	0.030255847	UP
208	P23526	GN=AHCY	0.06905619	1.640725017	0.002261663	UP
211	P08648	GN=ITGA5	0.295402576	2.49049753	0.039224207	UP
212	O43854	GN=EDIL3	0.222155178	2.968171537	0.014041941	UP
217	Q15758	GN=SLC1A5	0.217787312	2.194595277	0.022728641	UP

220	Q14697	GN=GANAB	0.278640637	3.214818001	0.023392667	UP
223	P10586	GN=PTPRF	0.019876031	1.545561016	7.54843E-05	UP
224	Q96TA1	GN=FAM129B	0.165982268	3.062427521	0.005919039	UP
228	P61764	GN=STXBP1	0.196213737	1.947686225	0.023210099	UP
229	Q92973	GN=TNPO1	0.046249609	2.160055757	0.00026867	UP
231	P26022	GN=PTX3	0.175602091	5.222239494	0.004114018	UP
233	Q92743	GN=HTRA1	0.147809661	0.126980398	4.21497E-06	DOWN
238	P07737	GN=PFN1	0.344810712	4.354136765	0.030536847	UP
240	P67775	GN=PPP2CA	0.320516746	3.369161963	0.032004747	UP
245	P35606	GN=COPB2	0.184328433	3.009013772	0.008175759	UP
246	Q7KZF4	GN=SND1	0.06880245	1.811476231	0.001495243	UP
249	Q99613	GN=EIF3C	0.076790575	2.043097734	0.00140585	UP
250	P09104	GN=ENO2	0.182987094	1.6914455	0.030540217	UP
251	P13929	GN=ENO3	0.025081467	3.236939549	2.02583E-05	UP
253	P23921	GN=RRM1	0.144378054	3.416344464	0.003437166	UP
260	P23396	GN=RPS3	0.043948006	1.529030263	0.000853234	UP
261	P84077	GN=ARF1	0.32933927	4.043222606	0.028783786	UP
262	O14745	GN=SLC9A3R1	0.304800275	4.350696146	0.022077809	UP
263	P09543	GN=CNP	0.2531781	8.25079155	0.009220392	UP
264	P27694	GN=RPA1	0.265259835	2.151910007	0.039615234	UP
265	P07947	GN=YES1	0.201538687	1.6936405	0.038923432	UP
266	B0I1T2	GN=MYO1G	0.328038991	2.817017257	0.042284319	UP
267	P35613	GN=BSG	0.23755372	2.893088341	0.017494268	UP
270	Q969P0	GN=IGSF8	0.159179141	1.631390482	0.024457632	UP
271	Q13228	GN=SELENBP1	0.105630234	1.859673023	0.004763215	UP
273	P15880	GN=RPS2	0.200262192	1.693931013	0.03827907	UP
274	P22102	GN=GART	0.122635082	1.82526049	0.007776124	UP
275	P06756	GN=ITGAV	0.147487422	1.55873546	0.024483775	UP
276	O00299	GN=CLIC1	0.224901836	2.524885535	0.018743477	UP
277	P23634	GN=ATP2B4	0.248236436	3.82361728	0.014171872	UP
278	Q00839	GN=HNRNPU	0.198641978	0.483755998	0.002627528	DOWN
279	P48163	GN=ME1	0.095211349	2.54654175	0.001588398	UP
283	Q06830	GN=PRDX1	0.240812305	5.208949268	0.010141424	UP
285	P53992	GN=SEC24C	0.068446485	1.538215995	0.003036915	UP
286	P11166	GN=SLC2A1	0.237793322	5.923529148	0.009040827	UP
288	Q4LDE5	GN=SVEP1	0.191267596	2.212808758	0.015706524	UP
289	P50395	GN=GDI2	0.178230604	1.820213974	0.022042311	UP
292	P25789	GN=PSMA4	0.232277697	2.300051719	0.024403734	UP
293	P01891	GN=HLA-A	0.048948271	1.83029148	0.000525818	UP
294	O75340	GN=PDCD6	0.089271425	4.51584959	0.00062986	UP
295	P54136	GN=RARS	0.037960381	2.015176535	0.000180362	UP
299	P49720	GN=PSMB3	0.264123673	2.38394475	0.031854117	UP
303	P20810	GN=CAST	0.278999545	3.712654769	0.02005662	UP

307	Q13740	GN=ALCAM	0.124483414	1.902786285	0.007075836	UP
308	Q16719	GN=KYNU	0.217562817	2.530990511	0.017055969	UP
311	P28074	GN=PSMB5	0.263902282	1.977263212	0.047710372	UP
320	P40925	GN=MDH1	0.096302546	2.512170017	0.001686034	UP
323	P27348	GN=YWHAQ	0.296408045	3.056894064	0.029293182	UP
327	P63241	GN=EIF5A	0.270017421	1.990902007	0.049610689	UP
328	O15067	GN=PFAS	0.054278021	1.818189979	0.000731957	UP
329	P26006	GN=ITGA3	0.222810301	3.132810473	0.013163043	UP
331	P09972	GN=ALDOC	0.261387766	3.507950306	0.017833163	UP
333	P50454	GN=SERPINH1	0.08739798	0.446339123	0.000147562	DOWN
334	P30101	GN=PDIA3	0.235184388	0.465965308	0.003490158	DOWN
336	P43490	GN=NAMPT	0.218263738	1.934349447	0.031294252	UP
337	P14868	GN=DARS	0.081137077	1.881206512	0.00212865	UP
338	P61225	GN=RAP2B	0.257384803	4.107569754	0.014647848	UP
352	P78310	GN=CXADR	0.232890567	2.72339803	0.018154408	UP
355	P38646	GN=HSPA9	0.085919111	2.221317798	0.001573338	UP
356	P62140	GN=PPP1CB	0.145088534	4.088369548	0.002879069	UP
357	Q99497	GN=PARK7	0.275961421	2.04944849	0.04880964	UP
358	Q15019	GN=SEPT2	0.192081308	1.863105774	0.024986922	UP
365	P28072	GN=PSMB6	0.15822946	1.739477515	0.018718241	UP
370	P12109	GN=COL6A1	0.048948141	0.282176144	3.02212E-06	DOWN
371	P37837	GN=TALDO1	0.137041563	2.280747741	0.005754237	UP
374	P61586	GN=RHOA	0.20185161	2.425737739	0.015031728	UP
375	Q15084	GN=PDIA6	0.089604124	0.224259526	7.371E-06	DOWN
380	Q9Y2T3	GN=GDA	0.284241571	2.443611771	0.036764632	UP
381	Q15907	GN=RAB11B	0.096288189	2.094232708	0.002551715	UP
385	O00391	GN=QSOX1	0.364319433	3.24573198	0.046096977	UP
393	Q92819	GN=HAS2	0.267083553	0.130799193	2.75007E-05	DOWN
394	P16152	GN=CBR1	0.153382333	2.399487734	0.007122717	UP
397	O60610	GN=DIAPH1	0.177687644	1.566534013	0.038771056	UP
404	Q99536	GN=VAT1	0.141798089	1.813053548	0.01196399	UP
405	P04114	GN=APOB	0.248156606	0.534312949	0.008919824	DOWN
406	Q01650	GN=SLC7A5	0.38472662	5.292524219	0.035457811	UP
407	P16144	GN=ITGB4	0.161272707	1.716890246	0.02068179	UP
408	P15121	GN=AKR1B1	0.220125024	2.652977735	0.016243978	UP
409	P04792	GN=HSPB1	0.159920621	2.257397741	0.009131364	UP
413	P68371	GN=TUBB4B	0.175080352	1.684357256	0.027654025	UP
414	P17302	GN=GJA1	0.20636872	0.104395911	5.90366E-06	DOWN
418	Q9HBH0	GN=RHOF	0.093295326	2.392717779	0.001695213	UP
419	Q8NFJ5	GN=GPRC5A	0.052081542	15.26194501	7.3201E-05	UP
423	O75390	GN=CS	0.135633336	2.123500943	0.006623592	UP
424	P36955	GN=SERPINF1	0.337814867	0.059061793	4.04415E-06	DOWN
425	Q13200	GN=PSMD2	0.272172093	2.658682823	0.028561262	UP

429	P84095	GN=RHOG	0.053049015	1.579813242	0.001250234	UP
430	P22234	GN=PAICS	0.142103022	2.325032532	0.006119515	UP
442	Q16790	GN=CA9	0.166651926	1.607336968	0.029386318	UP
446	P62269	GN=RPS18	0.057012732	1.801790476	0.000875277	UP
447	P08572	GN=COL4A2	0.103553674	0.541177139	0.00075971	DOWN
448	Q9Y5Y0	GN=FLVCR1	0.170296875	3.202165484	0.005999596	UP
453	P35222	GN=CTNNB1	0.195059949	1.691465259	0.035994885	UP
460	P67936	GN=TPM4	0.063947559	1.537759781	0.002494615	UP
463	O95084	GN=PRSS23	0.142515299	2.292969286	0.006360286	UP
467	P15559	GN=NQO1	0.122704744	1.951033741	0.00628787	UP
469	Q9BXX0	GN=EMILIN2	0.038275696	0.332136899	2.92589E-06	DOWN
470	P63208	GN=SKP1	0.345015446	4.053078175	0.032408163	UP
478	P12110	GN=COL6A2	0.345244257	0.263810318	0.000789141	DOWN
479	P15941	GN=MUC1	0.178541429	4.922299325	0.004501154	UP
484	Q14344	GN=GNA13	0.048442272	1.743853241	0.000612138	UP
485	P55290	GN=CDH13	0.054767217	0.607643947	0.000256756	DOWN
486	Q9UQP3	GN=TNN	0.445557315	0.280688951	0.002152244	DOWN
487	P50570	GN=DNM2	0.310733095	2.3465105	0.049384598	UP
488	P11169	GN=SLC2A3	0.244171959	6.745390892	0.009092564	UP
489	Q8WXI7	GN=MUC16	0.13703314	3.177971244	0.003236727	UP
492	Q02790	GN=FKBP4	0.255836894	2.521410763	0.026503546	UP
497	P05026	GN=ATP1B1	0.110403267	2.809333026	0.002064822	UP
500	Q9UKK9	GN=NUDT5	0.031398074	1.854439259	0.00013356	UP
501	P26599	GN=PTBP1	0.116537555	1.644210517	0.010085303	UP
511	P43034	GN=PAFAH1B1	0.132322057	2.635281205	0.003901084	UP
513	Q15274	GN=QPRT	0.103553798	2.892939985	0.001633028	UP
519	P02794	GN=FTH1	0.087951878	1.628095239	0.004731971	UP
523	P62745	GN=RHOB	0.202389568	2.192312509	0.018708831	UP
535	O00625	GN=PIR	0.234094924	2.172533751	0.028132353	UP
538	Q9ULF5	GN=SLC39A10	0.132866178	2.459161282	0.004493144	UP
542	P15529	GN=CD46	0.295195772	2.489530206	0.039180709	UP
544	Q9H444	GN=CHMP4B	0.151955083	0.526090145	0.001969671	DOWN
545	Q03169	GN=TNFAIP2	0.212643895	2.483523726	0.016583502	UP
546	O75347	GN=TBCA	0.128379516	1.988889784	0.006760321	UP
559	O15427	GN=SLC16A3	0.18678236	6.588417053	0.00428117	UP
567	Q01581	GN=HMGCS1	0.148586864	2.855834007	0.004772155	UP
571	P27797	GN=CALR	0.064776342	0.316892348	1.15036E-05	DOWN
575	P16949	GN=STMN1	0.169122707	2.548069954	0.008369085	UP
580	Q99471	GN=PFDN5	0.204802194	1.649304777	0.044737233	UP
583	P06858	GN=LPL	0.329128073	2.735262007	0.044434378	UP
589	P09382	GN=LGALS1	0.279049206	3.211142421	0.023516288	UP
591	P08253	GN=MMP2	0.199270046	0.369795024	0.000667533	DOWN
596	P23588	GN=EIF4B	0.226368322	1.746358722	0.046774006	UP

597	Q13085	GN=ACACA	0.141798318	1.969747961	0.009213613	UP
599	Q15818	GN=NPTX1	0.123789896	3.071518779	0.002522044	UP
608	Q92597	GN=NDRG1	0.062620133	1.509564489	0.002601586	UP
610	Q15008	GN=PSMD6	0.121554608	0.664086774	0.005505422	DOWN
614	Q76M96	GN=CCDC80	0.107800267	0.34004578	7.24625E-05	DOWN
617	Q14019	GN=COTL1	0.266786379	2.790770173	0.025168485	UP
619	Q13838	GN=DDX39B	0.346160998	0.574718714	0.034216979	DOWN
627	P46063	GN=RECQL	0.070009365	1.652202755	0.002281105	UP
634	Q9Y4D7	GN=PLXND1	0.106987901	2.02537775	0.003800679	UP
635	P29966	GN=MARCKS	0.277331136	2.044178009	0.049703489	UP
636	Q14643	GN=ITPR1	0.131674406	0.494053401	0.000884608	DOWN
646	Q13421	GN=MSLN	0.265216471	2.300362229	0.034475463	UP
649	P17813	GN=ENG	0.020593688	1.941297501	3.2445E-05	UP
650	P47712	GN=PLA2G4A	0.140402613	2.214427501	0.006598398	UP
655	Q9Y696	GN=CLIC4	0.247746712	2.578641742	0.023428869	UP
659	P02649	GN=APOE	0.283798251	0.483819611	0.007358071	DOWN
660	Q9ULV4	GN=CORO1C	0.081770964	1.881268263	0.002177498	UP
661	Q16181	GN=SEPT7	0.130717394	0.637865141	0.004868602	DOWN
664	P25815	GN=S100P	0.110950062	1.558434993	0.011284527	UP
666	Q86YQ8	GN=CPNE8	0.196296564	1.56479302	0.049910259	UP
685	P60033	GN=CD81	0.037459479	2.65646565	9.1614E-05	UP
688	O95163	GN=ELP1	0.025547223	1.832912266	7.51281E-05	UP
690	P61970	GN=NUTF2	0.121012815	1.542289495	0.015120304	UP
693	P26447	GN=S100A4	0.305414272	3.564374745	0.026588907	UP
700	Q9UM54	GN=MYO6	0.101773941	2.214436293	0.00260436	UP
701	P0C0L4	GN=C4A	0.443282985	0.466880582	0.020966473	DOWN
721	Q8WWI5	GN=SLC44A1	0.24484568	2.266194552	0.028896747	UP
722	P46782	GN=RPS5	0.177623227	1.901520491	0.01905265	UP
726	Q8IWA5	GN=SLC44A2	0.13078331	1.55494675	0.01794461	UP
728	P16444	GN=DPEP1	0.205932981	0.354338683	0.00060338	DOWN
730	P14174	GN=MIF	0.223463895	2.517688721	0.018514666	UP
738	Q9P0V9	GN=SEPT10	0.309237623	2.949533015	0.034228657	UP
739	P15144	GN=ANPEP	0.123096995	0.617046446	0.003161658	DOWN
742	P61769	GN=B2M	0.192473182	3.452935457	0.007752	UP
743	P14625	GN=HSP90B1	0.235532708	0.346729025	0.000813857	DOWN
747	P51153	GN=RAB13	0.100322924	1.600271016	0.007472246	UP
748	O60568	GN=PLOD3	0.319004638	0.568018109	0.025763869	DOWN
756	Q9UQB8	GN=BAIAP2	0.303054514	2.789776742	0.035081959	UP
760	P48668	GN=KRT6C	0.140433903	0.33060845	0.000140801	DOWN
765	P02792	GN=FTL	0.151957298	1.990992218	0.010849028	UP
773	O75462	GN=CRLF1	0.145448626	0.572254188	0.002990462	DOWN
775	P62826	GN=RAN	0.038074503	2.164292753	0.000149571	UP
779	P13987	GN=CD59	0.232372021	2.365009964	0.023110328	UP

786	P39019	GN=RPS19	0.250870787	2.452261239	0.026441285	UP
794	P10599	GN=TXN	0.185287439	1.756314754	0.027546924	UP
809	P62330	GN=ARF6	0.120959368	1.845780015	0.007199484	UP
814	O00592	GN=PODXL	0.196116018	2.114908218	0.0186931	UP
815	P21926	GN=CD9	0.071915869	1.824413002	0.001660172	UP
827	Q9Y224	GN=RTRAF	0.100954716	2.014712989	0.003260063	UP
828	Q13867	GN=BLMH	0.162126979	1.580280274	0.029468222	UP
835	Q9UKU9	GN=ANGPTL2	0.147378075	0.367886722	0.000265475	DOWN
846	Q9HA64	GN=FN3KRP	0.081960874	1.597566783	0.0042204	UP
852	P60953	GN=CDC42	0.248832039	1.865138024	0.048263246	UP
862	O43242	GN=PSMD3	0.006512646	1.659622252	1.86665E-06	UP
878	P06241	GN=FYN	0.131224188	1.555128485	0.018098858	UP
894	P55327	GN=TPD52	0.192054787	4.076690495	0.006485985	UP
895	P02458	GN=COL2A1	0.085116533	0.428126104	0.000109278	DOWN
948	Q14847	GN=LASP1	0.254220591	1.941772252	0.045581612	UP
949	P62879	GN=GNB2	0.097401983	3.483928025	0.001058378	UP
956	Q13283	GN=G3BP1	0.095761827	0.539501131	0.000590387	DOWN
957	P15311	GN=EZR	0.200577743	2.488340527	0.014078324	UP
959	P07108	GN=DBI	0.24538255	2.731844783	0.020802348	UP
995	P45877	GN=PPIC	0.078592407	0.467028759	0.000137843	DOWN
1010	Q96T76	GN=MMS19	0.081450138	0.665948495	0.001761057	DOWN
1025	O15078	GN=CEP290	0.394823153	0.354540247	0.004096398	DOWN
1032	O43847	GN=NRDC	0.086061502	1.661908001	0.004053501	UP
1047	Q96DG6	GN=CMBL	0.111624963	1.537166476	0.012306479	UP
1057	Q9UHD9	GN=UBQLN2	0.126795657	0.61025919	0.003170642	DOWN
1059	P42229	GN=STAT5A	0.130972458	1.624459982	0.014707071	UP
1062	P27658	GN=COL8A1	0.275552493	0.186448522	0.000106377	DOWN
1067	Q9H4G4	GN=GLIPR2	0.143968927	0.646434143	0.007141304	DOWN
1069	P08581	GN=MET	0.139628583	2.000989527	0.008432881	UP
1078	P62993	GN=GRB2	0.227707234	0.533742502	0.00694566	DOWN
1088	P60903	GN=S100A10	0.21825635	3.625924706	0.010463575	UP
1113	P98172	GN=EFNB1	0.16221356	0.545489013	0.002994795	DOWN
1135	P08476	GN=INHBA	0.192495199	0.559894986	0.005811605	DOWN
1150	P63096	GN=GNAI1	0.189277408	1.552084982	0.047309785	UP
1174	Q9UKG1	GN=APPL1	0.104969593	1.739388019	0.005951844	UP
1186	P29972	GN=AQP1	0.049062691	0.639028639	0.000275593	DOWN
1194	Q15012	GN=LAPTM4A	0.163638238	1.87420997	0.015935236	UP
1216	O00151	GN=PDLIM1	0.146920363	2.196431071	0.007653384	UP
1287	P06865	GN=HEXA	0.029486158	1.535283983	0.000254532	UP
1297	P19827	GN=ITIH1	0.154393251	0.615447491	0.005962681	DOWN
1298	P05121	GN=SERPINE1	0.260924271	0.548669741	0.012069309	DOWN
1306	P46459	GN=NSF	0.239231249	2.131202787	0.031088742	UP
2331	P63092	GN=GNAS	0.285662664	3.098692805	0.026140231	UP

2883	O00186	GN=STXBP3	0.223813527	2.248855501	0.023174829	UP
